# Supplementary figures and images for: Deciphering the involvement of the Hippo pathway co-regulators, YAP/TAZ in invadopodia formation and matrix degradation
Source: Cell Death Dis. 2023 Apr 25;14(4):290. doi: 10.1038/s41419-023-05769-1 (PMC10130049; doi:10.1038/s41419-023-05769-1)

**YAPTAZ Knockdown Blots**


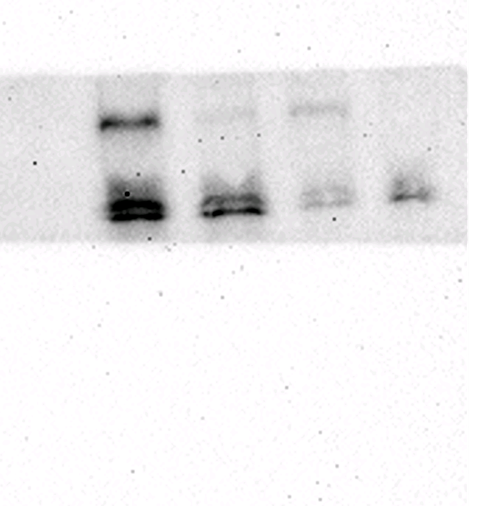


YAPTAZ


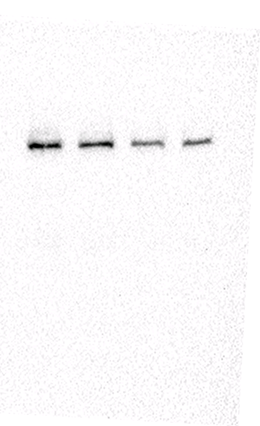


Vinculin

**YAPTAZ Overexpression Blots**

**
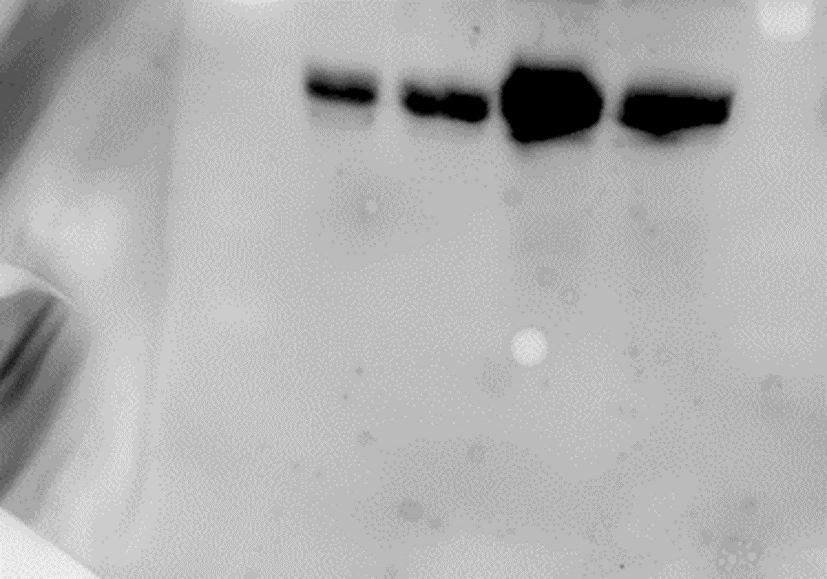
**

YAPTAZ

**
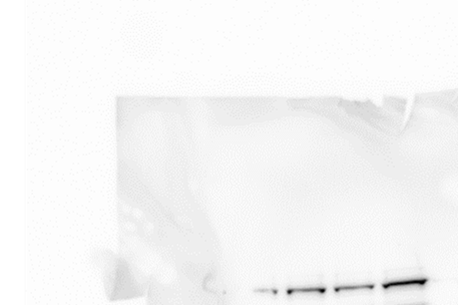
**

Flag

**
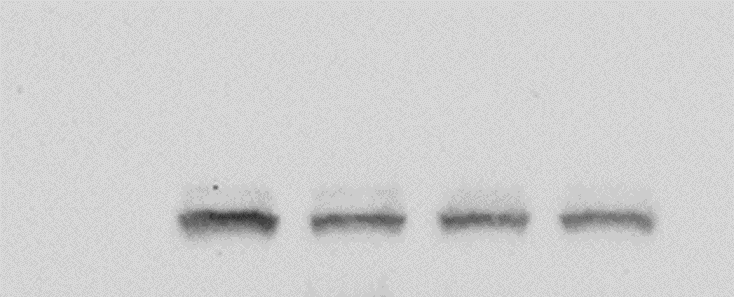
**

Vinculin

Supplement: Supplementary file 9 — Original Data File [file 41419_2023_5769_MOESM9_ESM.docx]
